# A Comprehensive Approach to Treating Epithelioid Hemangioendothelioma of the Auricle: A Case Report

**DOI:** 10.7759/cureus.95742

**Published:** 2025-10-30

**Authors:** George Yalamanchili, William J Smith, Nitin Vaishampayan, Yash Somnay, Sepideh Babaniamansour, Ehsan Aliniagerdroudbari, Kimberly B Hart, Steven R Miller

**Affiliations:** 1 Department of Oncology, Wayne State University School of Medicine, Detroit, USA; 2 Department of Pathology, Wayne State University School of Medicine, Detroit, USA; 3 Department of Oncology, Wayne State University, Detroit, USA; 4 Department of Radiation Oncology, Wayne State University School of Medicine, Detroit, USA

**Keywords:** adjuvant radiation therapy, chemotherapy, ent surgeries, epithelioid hemangioendothelioma, head and neck cancer pathology

## Abstract

Epithelioid hemangioendothelioma (EHE) is a rare vascular neoplasm that commonly presents in the bone, lung, and liver. EHE can have a slow-growing, locally aggressive, or metastatic potential. EHE of the auricle is rare, with few reported cases. Various treatment strategies have been reported on, but due to the rarity of EHE, their effectiveness has yet to be established. Surgical resection is the most common form of treatment, and adjuvant chemotherapy and radiation therapy have been performed in select cases. This report presents a case of auricular EHE, highlighting diagnostic challenges and therapeutic considerations. It aims to demonstrate the potential utility of adjuvant radiotherapy in managing EHE following surgical resection.

## Introduction

Epithelioid hemangioendothelioma (EHE) is a rare vascular tumor originating from vascular endothelial or pre-endothelial cells [1]. It is a malignant vascular tumor that can also be locally aggressive with metastatic potential [2]. Recurrent EHE can occur locally as well as distantly [3]. With an incidence estimated to be less than one in one million, EHE represents less than 1% of all vascular tumors [1]. EHE most commonly originates in the lungs, liver, or bones and, less frequently, may present in the head and neck region, breast, lymph nodes, brain, spine, abdomen, or skin [1]. EHE of the auricle can often be mistaken for an auricular pseudocyst [4], psoriasis, irritant dermatitis, and eczema [5]. EHE of the auricle is a very rare form of EHE, with only a few reported cases. EHE of the auricle can also involve the skin and subcutaneous tissue [6], the parotid gland [6], and lymph nodes [5]. Cutaneous EHE can be a primary lesion or result from a tumor that has metastasized to the skin. Due to its rarity and broad spectrum of possible primary sites, EHE is often misdiagnosed, highlighting the complexity of this tumor. Furthermore, the lack of established treatment guidelines adds to the challenge of managing EHE, with retrospective case reports or case series serving as the primary guidelines for treatment [2].

In this case report, we present a case of EHE involving the left auricle, involving the supra-auricular and pre-auricular area, status post surgery, adjuvant radiation, and chemotherapy.

## Case presentation

A female in her fourth decade of life with a history of depression, anxiety, migraines, back pain, and hearing loss initially presented to her dermatologist with a left ear supra-auricular and pre-auricular lesion that had been present for many years. She had been experiencing intermittent bleeding and an increase in size of both lesions for approximately one year.

Her past medical history included a 20-pack-year smoking history. Her family history is negative for malignancies. She denies a history of scleroderma, lupus, or other connective tissue disorders. She also denies having a pacemaker or automatic implantable cardioverter defibrillator (AICD) and has no history of prior radiation therapy.

She was referred to an otolaryngologist, and an evaluation at that time revealed a pre-auricular lesion approximately 3 x 2 cm in size, which was firm and flat with a cartilaginous stalk extending both anteriorly and medially. This was initially suspicious for a type one branchial cleft cyst. The supra-auricular lesion was noted to be a 1 x 1 cm firm mass without any appreciable extension into the surrounding tissue. 

**Figure 1:**
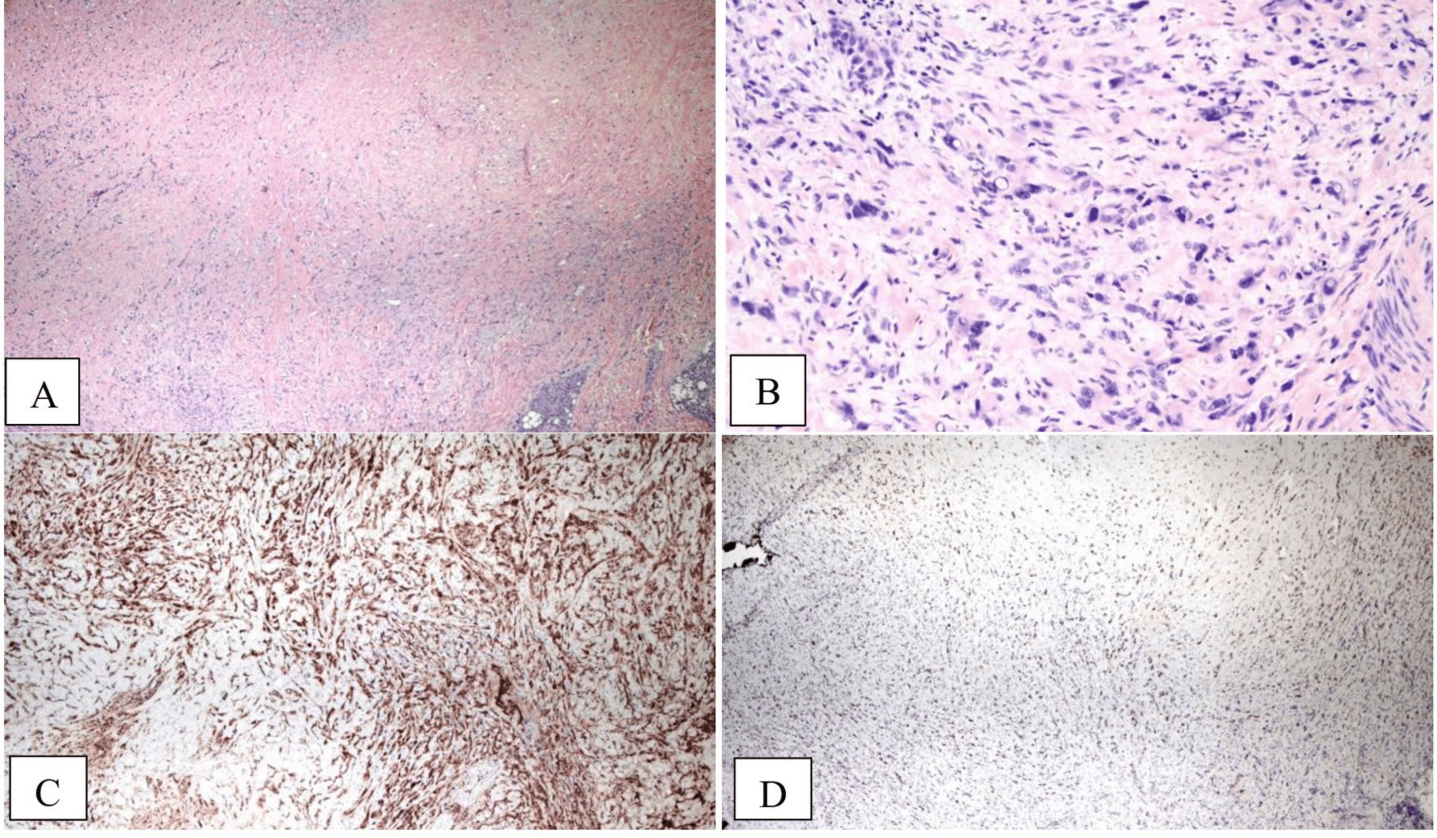
Microscopic examination of the left pre-auricular mass biopsy specimen revealed a vascular neoplasm composed of epithelioid to slightly spindled cells arranged in cords and small nests within a myxohyaline stroma (A, 4× magnification). The neoplastic cells showed moderate eosinophilic cytoplasm, round to oval nuclei with marked pleomorphism, and occasional intracytoplasmic vacuoles (B, 20× magnification). Mitotic activity was low, and no significant necrosis was identified. Immunohistochemical staining showed diffuse membranous staining for CD31 (C), supporting endothelial differentiation. Additionally, nuclear immunoreactivity for TFE3 (D) was present. These morphologic and immunophenotypic features are consistent with a diagnosis of epithelioid hemangioendothelioma.

She underwent a biopsy of the left pre-auricular lesion, and morphological characteristics of the surgical specimen revealed Intracytoplasmic vacuoles and mildly atypical epithelioid cells arranged singly, in cords, strands, or small nests, and embedded in a partly calcified myohyaline stroma (Figure 1).

Histological characteristics of the biopsy specimen included endothelial cells arranged in nests and cords, spindle-shaped tumor cells with various-sized lumens, and hyalinized stroma [1,5]. Cluster of differentiation 31 (CD31) is a particular vascular tumor marker for EHE, and Friend leukemia integration one transcription factor (FLI-1) is a transcription factor expressed in endothelial cells. The presence of these two markers is considered sufficient for an EHE diagnosis [1]. Transcription factor E3 (TFE3) is also consistently expressed and observed in immunohistochemistry [7]. The presence of CD31 and TFE3, along with the morphological and histological markers, was sufficient to diagnose EHE of the auricle [1,7,8]. 

Both masses and the cutaneous scar tissue were excised, with pathology demonstrating the following: Left pre-auricular mass, excision: EHE - 2.1 cm in size with tumor extending to the excision margins, and left supra-auricular mass, excision: EHE - 1.5 cm in size with tumor extending to the excision margins.

Pathological review of the surgical specimen confirmed the diagnosis of EHE. Immunohistochemical examination revealed that the specimens were positive for vimentin, CD31, and TFE3. Staining for CD31, a specific vascular tumor marker, along with nuclear immunoreactivity for TFE3, is consistent with the diagnosis of EHE [7,9]. CD34 and AE1/3 are expressed in some cases of EHE, but are not required for a diagnosis [9].

Postoperatively, the patient experienced sharp pain in the surgical bed and inner ear discomfort. She refused to undergo additional surgery. Her case was discussed in the multidisciplinary tumor board, and the recommendation of the tumor board was to offer her adjuvant radiation and chemotherapy secondary to the positive margin and the concern that she was at an increased risk of developing recurrent disease.

Six weeks post-surgery, imaging, including an MRI of the neck, revealed an ill-defined area of enhancement in the left temporal scalp and pre-auricular soft tissues. It was uncertain whether this represents postsurgical change versus a residual or recurrent tumor. No enlarged lymph nodes were noted. An MRI of the temporal bones and an MRI of the brainstem revealed persistent ill-defined enhancement in the left pre-auricular and supra-auricular surgical bed, likely representing a combination of residual tumor and postoperative change. There was no evidence of perineural disease involving the intratemporal left facial nerve.

She underwent an evaluation by radiation and medical oncology for concurrent therapy. The initial chemotherapy course consisted of Carboplatin and Taxol, but this was modified to Carboplatin monotherapy every three weeks following a hypersensitivity reaction to Taxol in the second week of treatment.

**Figure 2:**
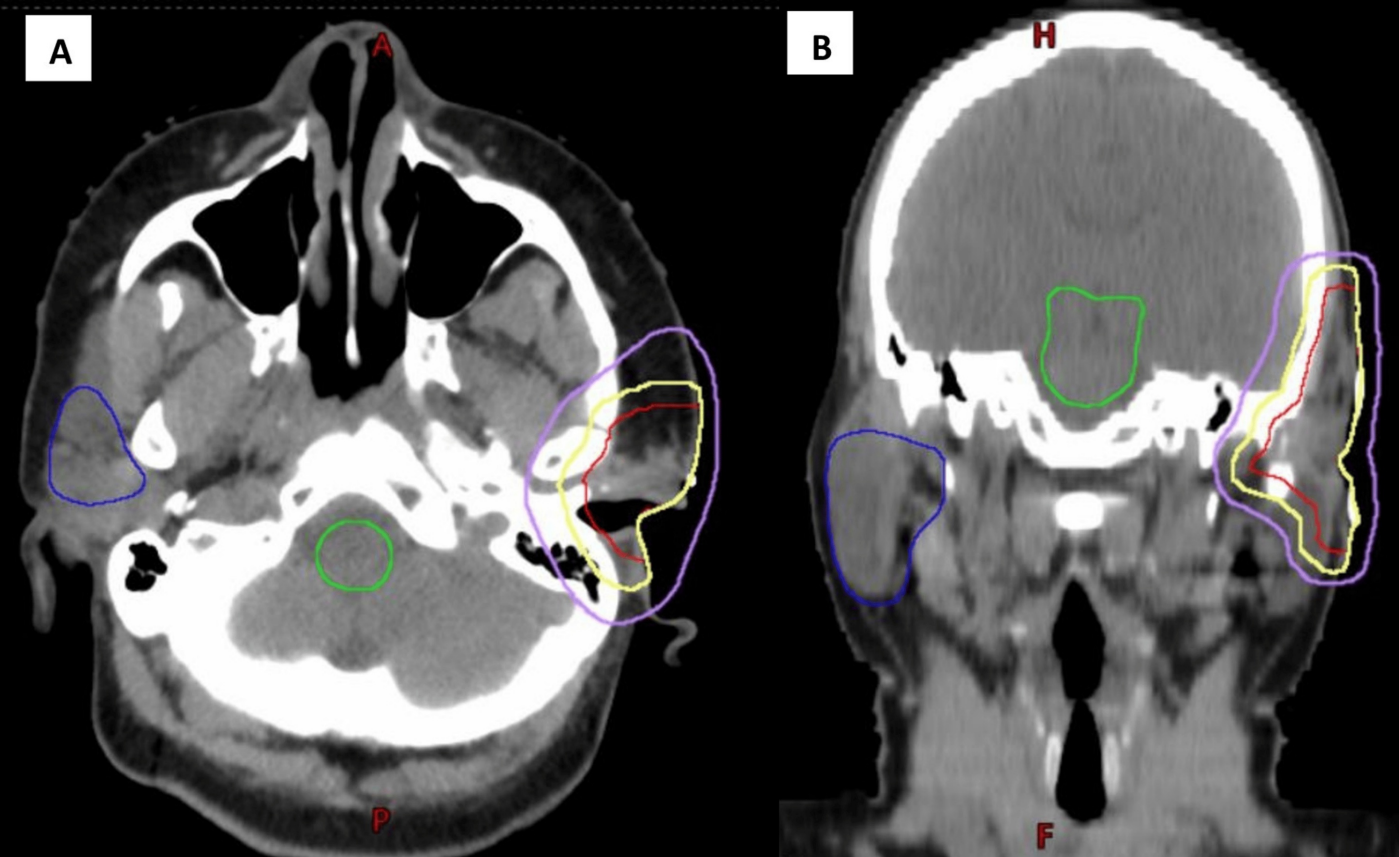
Axial (A) and coronal (B) images of the radiation therapy plan. Purple is the 4,750 cGy isodose line (95% of 5,000 cGy), red indicates the CTV5000, yellow the PTV5000, green the brainstem, and dark blue the right parotid gland.

The patient underwent a simulation in the radiation oncology department for planning radiation therapy. A planning CT scan was performed with the patient in the supine position, and a face mask was fashioned to stabilize the head and neck. The clinical target volume receiving 5,000 centigray (cGy) (CTV5000) consists of tissues considered at risk of harboring microscopic disease. The CTV5000 consisted of the surgical bed and post-surgical changes as identified on the planning CT scan. The planning target volume to receive 5,000 cGy (PTV5000) consists of the CTV5000 plus a margin to account for setup error and patient motion. The PTV5000 consisted of a 0.5 cm margin around the CTV5000, avoiding the skin surface. The PTV5000 was treated to a total dose of 5,000 cGy in 25 fractions using volumetric modulated arc therapy (VMAT; Figure 2). 

**Figure 3:**
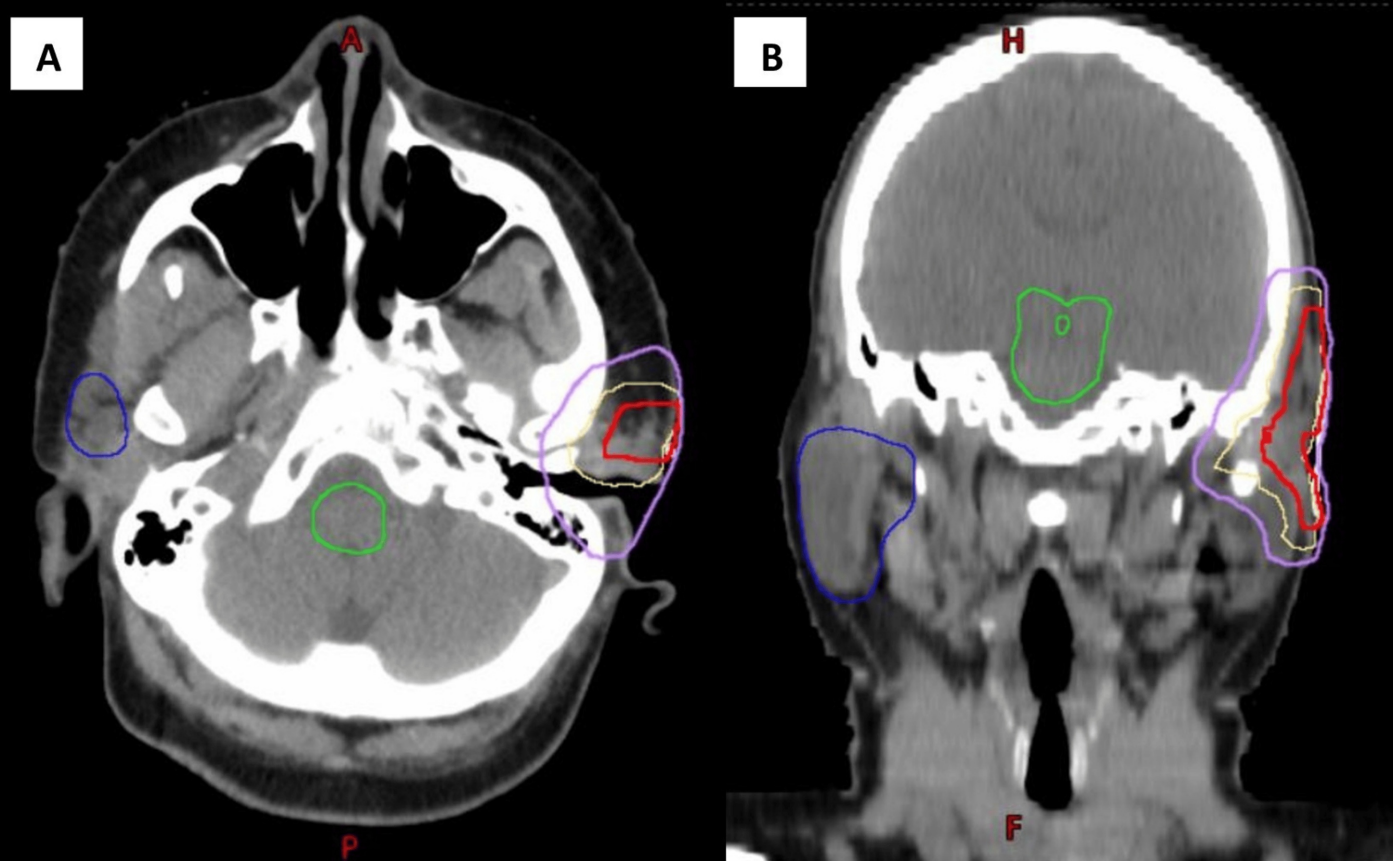
Axial (A) and coronal (B) images of the radiation therapy plan. Purple represents the 6,270 cGy isodose line (95% of 6,600 cGy); red indicates the CTV6600, yellow the PTV6600, green the brainstem, and dark blue the right parotid gland.

The CTV volume receiving 6,600 cGy (CTV6600) consisted of the tumor bed as delineated from the MRI and post-surgical CT scan. The PTV volume receiving 6,600 cGy (PTV6600) consisted of a 0.5 cm margin around the CTV6600 in all directions, avoiding skin. The patient received an additional 1,600 cGy for a total dose of 6,600 cGy to the PTV6600 using VMAT in 33 fractions (Figure 3).

The conformality index (CI) for the initial plan of 5,000 cGy was 1.25 and for the boost plan of 6600 cGy was 1.48.

**Figure 4:**
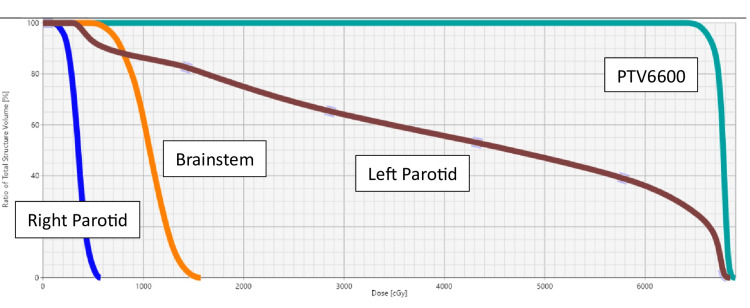
Dose-volume histogram (DVH). Dark green represents the PTV6600, brown the left parotid gland, orange the brainstem, and blue the right parotid gland.

Over 95% of the PTV6600 was covered by the prescription dose. The mean dose to the left parotid gland was 4,181 cGy, and the mean dose to the right parotid gland was 350 cGy. The maximum dose to the brainstem was 1,571 cGy. The doses to the following structures are displayed below in the dose-volume histogram (DVH) (Figure 4).

The patient tolerated treatment well, and two weeks after completing radiation and chemotherapy therapy, the skin of the left ear region had moderate hyperpigmentation with some mild radiation dermatitis. This was resolved within the next few weeks. 

A two-month follow-up revealed some mild radiation dermatitis and hyperpigmentation of the left ear. No physical evidence of recurrent disease was noted. A CT scan of the neck and chest, and a neck MRI obtained approximately four months status post surgery, radiation, and chemotherapy, revealed post-surgery and radiation changes with no enlarged lymph nodes in the neck and no evidence of recurrent disease.

## Discussion

E is a rare malignant vascular tumor that arises from endothelial or pre-endothelial cells. It is typically slow-growing, locally aggressive, and has metastatic potential [1,2]. EHE is typically seen in middle-aged adults with no known connection to the sex of the patient [10]. Patients with cutaneous EHE have a wide range of clinical presentations, posing a challenge to physicians. Some patients present asymptomatic, as in the two cases of auricular EHE reported by Wang et al. [4]. Symptomatic patients often report redness, scaliness, pain, and swelling [5,11].

According to reported cases over the past five years, treatment for auricular EHE typically involves surgical resection with adjuvant radiotherapy and chemotherapy, depending on the extent of the tumor [4,5,6]. Surgical resection can also be performed alone when the surgical margins are uninvolved and the risk of recurrence is low. Wang et al. report two cases of auricular EHE in which surgical resection was performed without adjuvant chemoradiation. At the six-month follow-up for the first patient and two-year follow-up for the second patient, no evidence of recurrent disease was identified [6]. In a case reported by Snyder et al., a patient received adjuvant radiotherapy after surgical resection. At follow-up, no tumor recurrence was noted [4]. Notably, these case reports emphasized the need for frequent follow-ups and surveillance imaging due to the risk of recurrence [5]. 

While surgical excision remains the mainstay of treatment for soft tissue EHE, no standardized management algorithm exists due to its rarity and unpredictable clinical behavior [1]. Chemotherapy is often used in cases of widespread disease [1]. Carboplatin [12] and Taxol [13] have been reported to achieve partial and near-complete responses when used in the metastatic setting. Radiation therapy is used in the treatment of soft tissue EHE based on guidelines for soft tissue sarcomas [2]. EHE is considered radiosensitive, demonstrating a response to doses of 3,000-4,000 cGy [3]. Radiotherapy is considered when lesions are unresectable, recurrent, or associated with involved surgical margins [1,2]. Local recurrence in EHE following surgical resection ranges from 10% to 15% [2]. A dose of 6,000 cGy delivered in 30 fractions is generally recommended in cases with positive margins or when there is significant concern for local recurrence [2].

Deyrup et al. proposed a risk stratification for EHE that involved two groups: the high-risk group had a tumor size greater than 3 cm and mitotic activity greater than three mitotic events per 50 high-power fields [14]. Anything below these values was considered part of the low-risk group. In their study of 46 patients with soft tissue EHE were retrospectively evaluated, most of these patients had a complete surgical resection performed. Patients in the high-risk group had the worst prognosis with a five-year disease-specific survival of 59%. None of the patients with low-risk features died [14]. The overall five-year disease-specific survival was 81%.

Due to the similar clinical presentation of auricular EHE to other auricular abnormalities, such as auricular pseudocysts [4], psoriasis, irritant dermatitis, or eczema [5], and in this case, a type one branchial cleft cyst, EHE diagnosis is often established pathologically after surgical excision. Alternatively, if vascular proliferation is suspected, a deep shave or punch biopsy can be performed at initial presentation, enabling the providers to diagnose and address treatment options earlier [5], which could improve local control and possibly survival.

Aksoy et al. reported a case with an unresectable EHE lesion involving the left supraclavicular region. The patient presented with neck pain and a palpable mass. She was treated with intensity-modulated radiation therapy (IMRT) to a total dose of 6,000 cGy in 30 fractions, targeting the gross tumor volume identified on magnetic resonance imaging (MRI) and fluorodeoxyglucose positron emission tomography (FDG-PET), with an additional margin. At a three-month follow-up visit, imaging showed a complete response, and the patient remained symptom-free on subsequent follow-up visits [15].

Wang et al. present two cases of auricular EHE that were initially diagnosed as auricular pseudocysts in a 48-year-old and a 65-year-old male. The tumors were surgically removed with negative margins with no adjuvant treatment. No evidence of recurrent disease of either of the tumors was noted at follow-up. This report acknowledges the utility of adjuvant radiotherapy. Still, it raises questions about its effectiveness in treating EHE, necessitating further clinical studies to gain a better understanding of the role of adjuvant radiation in EHE treatment [4].

Snyder et al. report a case of a 41-year-old female with EHE of the auricle and metastasis to a nearby lymph node: an auriculectomy, temporal bone resection, and left-sided neck dissection were performed in an attempt to render the patient disease-free. Metastatic disease was found in 1 of 12 lymph nodes, and the deep margin of the superior aspect of the external auditory canal was involved. She underwent adjuvant radiation therapy to a total dose of 6,600 cGy to the left neck resection bed, which was tolerated well [5].

Senaratne et al. reported a case of an 81-year-old male diagnosed with auricular EHE with a mitotic index of up to 6/10 high-powered fields, indicating a high-risk tumor. The patient underwent an excisional biopsy followed by reconstruction with a trapdoor flap. Subsequently, a CT scan revealed a soft tissue enhancement above the left auditory canal involving the skin and subcutaneous tissue, along with involvement of the parotid gland. The patient underwent a further wide local excision, and the margins were found to be negative. The patient underwent adjuvant radiotherapy [6]. The authors concluded that in the case of malignant cutaneous EHE, adjuvant radiotherapy plays a crucial role in preventing the disease from recurring and spreading to lymph nodes or other nearby structures, such as the auricle. This case highlights the importance of continuous monitoring in the care of a patient with EHE of the earlobe, especially when the tumor presents as high risk [6].

Soni et al. reported a case of right submandibular gland EHE involving bone, one lymph node, and metastasis to bilateral lungs. The patient underwent a right segmental mandibulectomy with bilateral supraomohyoid neck dissection. After surgical resection, histopathology indicated tumor involvement of the right submandibular gland and nearby bone. Positive margins were present, and one lymph node on the left side was positive for malignancy. The patient was initially treated to a dose of 5,000 cGy in 25 fractions to the tumor bed and bilateral neck region. This was followed by a 1,000 cGy boost in five fractions to the postoperative bed and involved the lymph node region. She also received cisplatin chemotherapy during radiotherapy. Grade 1 dermatitis and grade 2 mucositis were reported, but the patient recovered well from acute toxicities two weeks after the completion of concurrent chemoradiotherapy. The patient was then started on pazopanib. After three months, submandibular fibrosis was noted with stable disease in the lungs. After two years, the patient remained asymptomatic, and PET-CT scans indicated regional control without any metabolically active lung metastases [16].

Lee et al. reported a case of multiple cutaneous EHE on both feet of an 81-year-old female treated successfully with definitive radiotherapy. The patient underwent VMAT and received a total dose of 4,000 cGy over 20 daily fractions. The RT field involved her foot dorsum and entire sole, which began to develop edema and dermatitis. There was a break of two months before delivering an additional 2,000 cGy for residual disease. Four months later, lesions on the soles were resolved, and only the lesion on the foot dorsum remained. After seven months, the lesions were almost completely resolved. Twenty months after treatment, there was no evidence of disease recurrence [3].

## Conclusions

EHE is a rare vascular neoplasm for which there are no standardized treatment guidelines. Treatment can include a combination of surgery, chemotherapy, or radiotherapy. Close follow-ups and surveillance imaging are necessary in EHE to evaluate for recurrent disease. This case highlights the importance of early evaluation for patients with suspected auricular EHE and underscores the value of multidisciplinary management, including surgery, radiation, and chemotherapy, in optimizing patient outcomes.
